# The Effect of Microstructural Changes on Mechanical and Electrochemical Corrosion Properties of Duplex Stainless Steel Aged for Short Periods

**DOI:** 10.3390/ma13235511

**Published:** 2020-12-03

**Authors:** David D. S. Silva, Lindolpho S. D. C. Lima, Allan J. M. Araújo, Vinícius D. Silva, Rafael A. Raimundo, Igor Z. Damasceno, Thiago A. Simões, Rodinei M. Gomes

**Affiliations:** 1 Graduate Program in Mechanical Engineering, Federal University of Paraíba, João Pessoa 58051-900, Brazil; rar@academico.ufpb.br (R.A.R.); rodinei@ct.ufpb.br (R.M.G.); 2 Graduate Program in Materials Science and Engineering, Federal University of Rio Grande do Norte, Natal 59078-970, Brazil; lindolpho.lima@bct.ect.ufrn.br (L.S.D.C.L.); allanmenezes@ufrn.edu.br (A.J.M.A.); igorzumba@ufrn.edu.br (I.Z.D.); 3 Graduate Program in Materials Science and Engineering, Federal University of Paraíba, João Pessoa 58051-900, Brazil; vinicius.silva@ct.ufpb.br; 4 Center for Science and Technology in Energy and Sustainability, Federal University of Recôncavo da Bahia, Feira de Santana 44042-280, Brazil; thiago.simoes@ufrb.edu.br

**Keywords:** duplex stainless steel, sigma phase, electron backscatter diffractometry, mechanical properties, corrosion, electrochemical impedance spectroscopy

## Abstract

This work reports the effects of Microstructural changes due to the secondary phases, in particular sigma (*σ*), on the mechanical properties and electrochemical behavior of thermally aged duplex stainless steel (DSS). Structural, morphological, mechanical, and electrochemical characterizations were performed. Sigma phase content increased with increasing aging treatment time. It had a net-like shape, as observed by electron backscatter diffractometry (EBSD). Its presence directly damaged mechanical properties. The corrosion assessment included electrochemical impedance spectroscopy (EIS) in 1 M NaCl solution at temperatures of 25, 40, and 65 °C. EIS results demonstrate that an increase in the *σ* phase content decreased the corrosion resistance (21.1–0.8, 3.5–0.3, and 3.1–0.2 kΩ cm^2^ at 25, 40, and 60 °C, respectively).

## 1. Introduction

Duplex stainless steels (DSSs) are ferritic–austenitic steels with ferrite (*α-Fe*) and austenite (*γ-Fe*) volume fractions of about 1:1, providing an attractive combination of corrosion resistance and mechanical properties suitable for use in aggressive environments [1]. These properties, in combination with the effects of grain refinement obtained by the biphasic structure and the hardening by solid solution, has stimulated the use of DSSs in petrochemical, nuclear, cellulose industries, among others [2]. In addition, duplex alloys are often more resistant to corrosion and have superior mechanical properties to most common austenitic stainless steels, and they often overwhelm the performance of their austenitic counterparts. The ferritic structure confers high mechanical strength with high chloride-corrosion and stress-corrosion-cracking resistance, while the austenite renders high toughness with good resistance to hydrogen-induced stress-corrosion cracking [3,4].

The importance of controlling the microstructure of stainless steels in order to improve their material response is not only limited to duplex stainless steel. Escobar et al. [5] investigated the stabilization mechanism and austenite reversion during inter-critical tempering at 625 °C of a Ti-stabilized supermartensitic stainless steel (SMSS). The authors concluded that the austenite equilibrium phase fraction was reached after 2.5 h of isothermal reversion. Nonetheless, compositional homogenization was not reached within this soaking time. Escobar et al. [6] also studied the meta-equilibrium transition microstructure in a Ti-stabilized SMSS for maximum austenite stability and minimum hardness. The mechanism that explains the gradual increase in stable reverted austenite was the generation of a meta-equilibrium state, which resulted in a limit in both room-temperature austenite stabilization and high-temperature austenite reversion. However, maximizing reverted austenite and suppressing fresh martensite at room temperature did not lead to further reductions in hardness.

Despite all its advantages, DSS, when exposed to temperatures between 250 and 1000 °C, generates the precipitation of undesired phases through casting, rolling, welding, forging, and aging processes that affect corrosion resistance and mechanical properties [7,8]. When the steel is exposed to temperatures between 250 and 550 °C, the main reaction is related to the alpha-prime phase (*α’*) precipitation by a spinodal decomposition mechanism [9]. Secondary phases (e.g., sigma (*σ*), chi (*χ*), secondary austenite (*γ*_2_), as well as precipitates of chromium nitrides and carbides) are precipitated between 600 and 1000 °C, and depending on the temperature to which the material is exposed, the sigma phase can appear after 5 min [10,11,12,13,14,15,16,17,18].

The surface oxide film on DSSs, called a passive film, can effectively hinder further interaction between the metal and its environment, particularly when the film is generated in aqueous environments [19]. The corrosion resistance of DSS depends strongly on the properties of the passive film and the alloying elements involved in surface oxidation [20]. Therefore, any factor that causes surface defects in passive film or chemical or physical heterogeneity, such as inclusions, second-phase particles, solute-segregated grain boundaries, flaws, mechanical damage, or dislocations, is considered a possible reason for a decrease in corrosion resistance [21].

The kinetics of *σ* phase precipitation is controlled by the diffusion of Cr and Mo [22]. Regarding the *γ*_2_ phase, Cr and Mo contents in *α*-Fe decrease and Ni contents increase simultaneously after the precipitation of the *σ* phase. The *χ* phase it presents a remarkably higher amount of Mo than any other phase present, and a similar Cr content to that of the *α*-Fe phase [14,23]. During precipitation of these secondary phases, the elements responsible for DSS corrosion resistance are removed from the surroundings of these phases [7,24]. The precipitation of sigma phase enriched in Cr in the ferrite of DSS causes the segregation of the Cr concentration on the alloy surface. The higher the difference in Cr content between the *σ* and *γ*_2_ phases (a Cr-depleted zone), the worse the heterogeneity of the passive film and the poorer the resistance to pitting corrosion [21]. Therefore, the mechanical and corrosion properties of DSS are significantly modified by metallurgical variables [25,26].

The effect of the sigma phase on mechanical properties deserves to be studied, since a small fraction of this phase affects ductility and toughness [27,28]. Recently, Silva et al. [29,30] investigated the use of an electromechanical impedance (EMI) technique and low-field magnetic analysis (LFMA) in the monitoring of sigma phase embrittlement in DSS, in order to assess modifications in the microstructure of thermally degraded 2205 DSS at 800 °C for different time intervals. The authors confirmed that small *σ* contents (about 6%) promoted a considerable decrease of approximately 63% of toughness in DSS.

The influence of the sigma phase on the corrosion resistance of DSS has been reported as one of the major concerns of DSS welding [31,32,33]. Nonetheless, the characterization of DSS by directly linking microstructure (using electron backscatter diffractometry (EBSD)) to provide various structural and morphological data, and the relationship with electrochemical measurements, seems to be neglected in the literature.

In this regard, this work reports the use of EBSD along with electrochemical impedance spectroscopy (EIS) in order to understand the influence of secondary phases (mainly *σ* phase precipitation) on the corrosion resistance of DSS. Furthermore, the effects of *σ* phase on mechanical properties were studied by microhardness and tensile tests. For this, isothermal aging treatment at 800 °C was performed for different durations (15–180 min). The temperature and periods of heat treatment were chosen in order to simulate processes such as welding and thermal aging, to which these materials may be exposed.

## 2. Materials and Methods

The specimens used in this investigation were commercial duplex stainless steel SAF2205. The chemical composition (in wt.%) of the duplex stainless steel according to the ASTM A240/A240M-20 [34] was: C (0.025), Si (0.31), Mn (1.52), P (0.021), S (0.001), Cr (22.15), Mo (3.16), Ni (6.20), Cu (0.15), N (0.176), and Fe (Bal.).

### 2.1. Heat Treatment

A solubilization heat treatment was carried out at 1155 °C for 1 h. Then, an isothermal aging treatment at 800 °C was performed for 15, 30, 60, and 180 min. A specimen which was only solubilized (without aging treatment, 0 min) was also used. The cooling was done by water quenching.

### 2.2. Structural, Morphological, and Mechanical Characterization

X-ray diffraction measurements were carried out within a scan range of 41.5–52.5° (2-theta), with a step size of 0.02° and 1 s per step by a diffractometer (SIEMENS D5000, Munich, Germany) equipped with Cu radiation (Kα, λ = 1.5418 Å). XRD patterns were compared to the card files: *α*-Fe phase (ICSD n° 625865), *γ*-Fe phase (ICSD n° 53803), *σ* phase (ICSD n° 102759), and *χ* phase (ICSD n° 102758).

Microstructural examination of the specimens was conducted using a light optical microscope (Olympus BX41M-LED, Tokyo, Japan). The specimens were polished by diamond paste (6–0.25 μm) and then electrolytically etched by a 40% NaOH electrolyte at 3 V etching potential for 30 s, according to ASTM A923-14 [35].

Electron backscatter diffraction (EBSD) phase analysis was also employed. Metallographic polishing of specimens for EBSD measurements was carried out by standard preparation methods using diamond pastes with particle sizes down to 0.25 μm. In addition, residual stresses caused by the earlier stages of mechanical polishing were relieved by a final polishing step using a suspension of colloidal silica (0.05 μm). Zeiss AURIGA field emission gun SEM (FEG-SEM, Zeiss, Oberkochen, Germany) equipped with a BRUKER e-FlashHR EBSD detector (Bruker, Billerica, MA, USA) was used. Step sizes were chosen between 47 and 230 nm and the voltage was 20 kV. ESPRIT 2.2 software (Bruker) was used to generate inverse pole figures (IPFs) and phase maps combined with the image quality (IQ). The EBSD area fractions of phases were obtained from the mean of three measurements.

**Figure 1:**
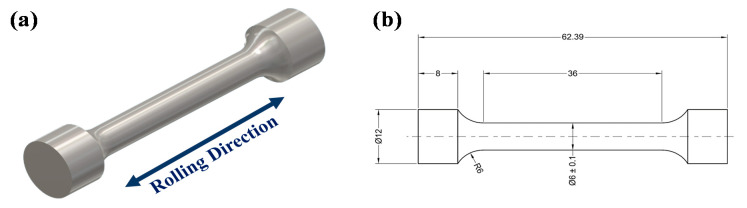
(**a**) Schematic illustration of the tensile specimens and (**b**) dimensions in mm of an ASTM-A370-20 (2020) test specimen.

The hardness test was conducted using a Vickers Micro Hardness Tester (Shimadzu HMV, Kyoto, Japan). The applied load and holding time were 200 g and 15 s. The tensile test was performed by a Shimadzu AG-100KN electronic universal testing machine (Kyoto, Japan). Cylindrical specimens (Figure 1) were elongated at a constant cross-head speed of 2 mm/min according to the ASTM-A370-20 [36] test method. The fractured surfaces of the specimens after testing were analyzed using the LEO 1430 scanning electron microscope (Carl Zeiss AG, Jena, Germany).

### 2.3. Electrochemical Corrosion Characterization

EIS studies were performed using a PGSTAT204-FRA32M (Metrohm Autolab, Utrecht, The Netherlands). A three-electrode cell (volume 100 mL) in naturally aerated conditions was used. DSS specimens with different heat treatments were used as a working electrode (leaving an exposed area for corrosion test of 0.80 cm^2^) and further sanded on abrasive papers with grain size ranging from 220 to 600 µm/mm^2^. Platinum wire and Ag/AgCl (3 M KCl) were used as counter and reference electrodes, respectively. Before starting the EIS tests, the specimens were passivated using an aqueous solution containing 20% HNO_3_ at 40 °C for 20 min.

All EIS experiments were performed in a 1 M NaCl solution at temperatures of 25, 40, and 65 °C (controlled using a hot plate and thermometer). Before testing, the working electrodes were immersed in the test solution for 30 min until steady-state open-circuit potential (OCP) was reached. EIS was carried out at the OCP to electrochemically characterize the specimen/electrolyte interface. EIS measurements were carried out in the frequency range 1 MHz–1 Hz using a voltage amplitude of 5 mV.

Roughness measurements by optical profilometry were conducted using a CCI MP Taylor Hobson^®^ profilometer (Taylor Hobson, Leicester, UK). The results were analyzed using TalyMap^®^ 5 software (Taylor Hobson). Specimens were magnified (×50) with a resolution of 1024 × 1024 pixels. The 3D topography of the surface was acquired by combining several measurements.

## 3. Results and Discussion

### 3.1. Structural, Morphological, and Mechanical Characterization

**Figure 2:**
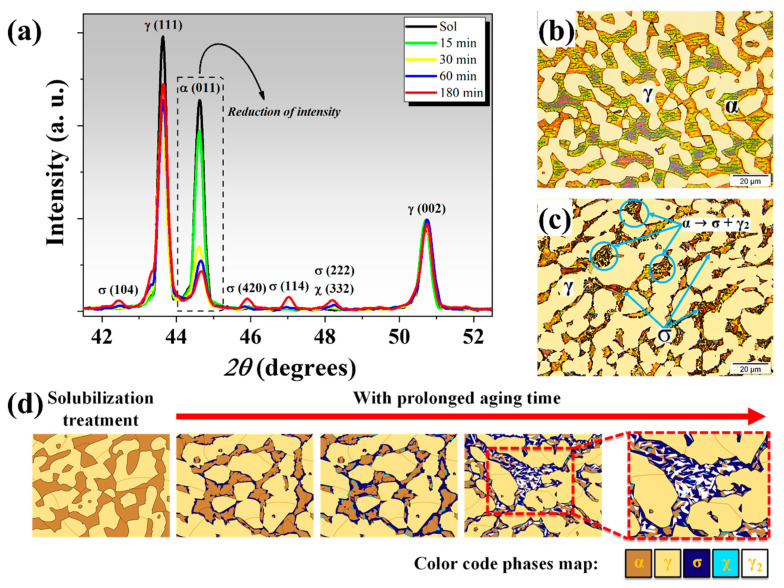
(**a**) XRD patterns, (**b**,**c**) OM of the specimens (0 and 180 min of aging, respectively), and (**d**) a schematic illustration of the microstructural evolution of DSS with prolonged aging time.

XRD patterns of all the specimens are shown in Figure 2a. The solubilized specimen had no intermetallic phases, and only characteristic peaks of *α*-Fe and *γ*-Fe phases were identified. However, *χ* and *σ* phases were also identified for the aged specimens. Note that the *χ* phase diffraction peak (332) is coincident with the *σ* phase peak (222). The intensity of *σ* phase peaks increased with increased aging time, accompanied by a reduction in the intensity of the *α*-Fe phase peak (011).

Figure 2b,c displays the microstructures for the solubilized specimen, and for the one aged for 180 min. The solubilized specimen only contained *α*-Fe and *γ*-Fe phases, while formation of the *σ* phase could be seen after aging. The precipitation of the *σ* phase led to the formation of *γ*_2_ in a process defined as eutectoid decomposition (*α → σ + γ*_2_); as seen in Figure 2c. These results are in agreement with previously studies [29,30].

These optical microscopies (OM) show that the secondary phases grew at the expense of the *α*-Fe phase. However, these results do not provide a good contrast for distinguishing between the various secondary phases, mainly the *σ*, *χ*, and *γ*_2_ phases. In order to clarify the precipitation behavior and phase transformation of DSS, a schematic illustration is shown in Figure 2d.

**Figure 3:**
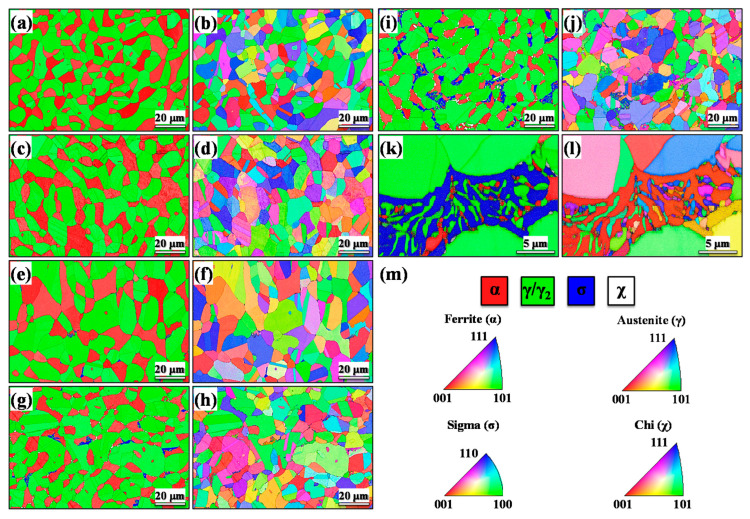
Electron backscatter diffractometry (EBSD) phase map of the specimens in the following conditions: (**a**) solubilized and aged for (**c**) 15 min, (**e**) 30 min, (**g**) 60 min, and (**i**,**k**) 180 min. Inverse pole figure (IPF) orientation maps of the specimens in the following conditions: (**b**) solubilized and aged for (**d**) 15 min, (**f**) 30 min, (**h**) 60 min, (**j**,**l**) and 180 min; (**m**) color code map for IPF [001]. Note that the aged specimen for 180 min has two images (medium and high magnification).

Considering that it is almost impossible to differentiate sigma and chi phases using the XRD and OM techniques, especially in the earlier stages of aging, electron backscatter diffractometry (EBSD) analysis was performed. Figure 3 shows the EBSD phase maps and inverse pole figure (IPF) maps. The contrast on these maps provides useful visualization of grain boundaries, permitting the alignment of different images. These results illustrating the distribution and morphology of phases which precipitating at different aging times. The maps clearly show the distribution of the different phases in duplex stainless steel.

Note that the initial microstructure (Figure 3a) consisted only of *α*-Fe and *γ*-Fe phases. However, the microstructures of the aged specimens in Figure 3c,e,g,i (15, 30, 60, and 180 min aging, respectively) showed a fraction of secondary phases (*σ*, *χ*, and *γ*_2_) which formed during the aging treatment [27]. These results are in agreement with the XRD and OM results. A high-magnification EBSD map (Figure 3k) allowed us to observe the microstructure of specimens aged for 180 min. *γ*_2_ formed together with other secondary phases associated with eutectoid *σ* phase formation in the ferrite, resulting in elongated net-like shapes. *χ* precipitates were seen with an elongated shape. *σ* phase had preferential growth along the phase boundaries with an allotriomorphic morphology towards ferrite grain interiors [37]. Based on the IPF maps, no specific texture was observed, and these phases were found to be randomly distributed. Similar behavior was previously reported [38].

Quantitative analysis of phases measured by EBSD is shown in Table 1. For each specimen, three EBSD images were obtained, the average of three measurements was reported, and the errors were based on the standard error of the mean. With the measurement methodology applied, the relative error of the area fraction evaluation of phases did not exceed 2.5%. The area fractions of the secondary phases increased with the increase of the heat treatment time. The *σ* phase increased from 0.4% to 6.8% and the *χ* phase increased from 1.2% to 2.3%, resulting in a decrease in the *α*-Fe phase from 37.5% to 17.2% with increasing aging time. The overall fraction of the *γ*_2_ phase formed was about 11.2%, which was determined by the difference between the content of the specimen aged for 180 min and the solubilized specimen. These results are following those previously reported [37,39].

**Figure 4:**
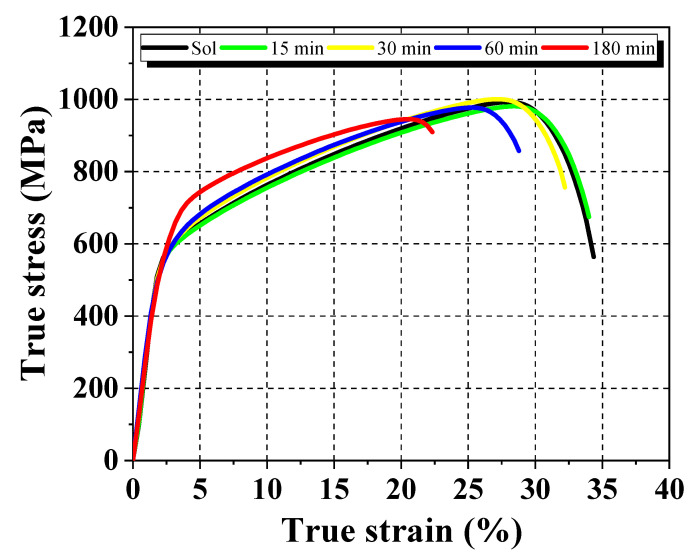
True stress versus true strain behavior as a function of aging time at 800 °C.

The influence of the *σ* phase on the mechanical properties of the DSS was examined by conducting tensile and microhardness tests. Figure 4 shows the typical stress versus strain curves for the UNS S31803 DSS specimens determined from uniaxial tensile test data. A summary of the results of the tensile test and the microhardness test are shown in Table 2. The microhardness (HV_0.2_) value increased by around 19%, resulting in a reduction of about 35% in strain (*ε*), showing clear signs of sigma phase embrittlement. The increase in *σ* content led to an increase in the yield strength (*σ_y_*), with values ranging from 507 to 561 MPa. However, notice that the ultimate tensile strength (*σ_u_*) was almost the same for all the specimens with a variation less than 5.5%. In general, the reduction in cross-sectional area (%RA) is a characteristic which describes the ductility together with the tensile test data. The solubilized specimen had a large %RA, about 57%, while the specimen aged for 180 min exhibited a %RA below 15%, showing a brittle behavior. This is due to the increased amount of the *σ* phase [27,29].

**Figure 5:**
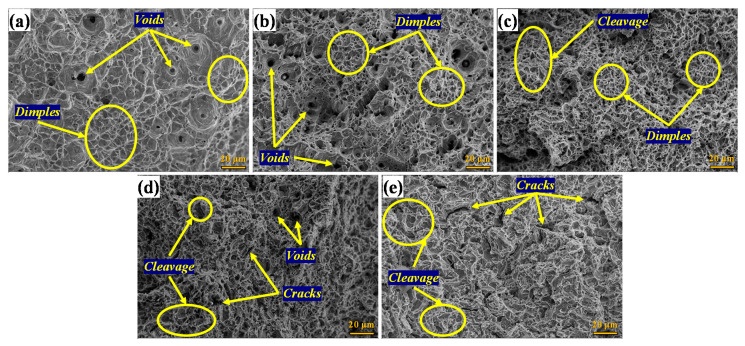
(**a**–**e**) SEM of fracture surfaces of the specimens (0–180 min, respectively).

Figure 5 shows SEM images of the surfaces of tested materials. Figure 5a shows the fracture surfaces of the solubilized specimen with a characteristic ductile fracture mechanism, wherein a large number of deep dimples and voids can be observed. Figure 5b shows the fracture morphology of the specimen aged for 15 min. The ductile fracture mode is evident again, with significant heterogeneous-size dimples. The specimen aged for 30 min (Figure 5c) shows the mixed mode of ductile and brittle fracture morphology. Figure 5d,e shows the fracture morphology of the specimens aged for 60 and 180 min, respectively. More extensive cracking was observed with significantly less necking and fewer dimples—all indicating embrittlement. Cleavage fracture areas were observed, which indicated strain heterogeneity in the microstructure. The presence of the *σ* phase can cause localized tearing that forms cracks. These results are in accordance with those previously reported [27,28,29,40].

### 3.2. Electrochemical Corrosion Tests

The surface roughness of a metal has a major influence on corrosion resistance. An increase in the surface roughness of stainless steels results in an increase in pitting susceptibility and in the general corrosion rate [41]. For the case of DSS, which form a passive layer, a decrease in surface roughness increases corrosion resistance [42]. Therefore, taking into account that the initial roughness can influence the corrosion resistance of the material, roughness measurements are necessary to prove that all specimens were subjected to the corrosion test with similar surface roughness, thus ensuring that only the precipitation of secondary phases influences the corrosion resistance, as well as the temperature of the electrochemical test. 2D and 3D surface roughness profile results (Figure S1) and further details on this characterization can be found in the Supplementary Material.

**Figure 6:**
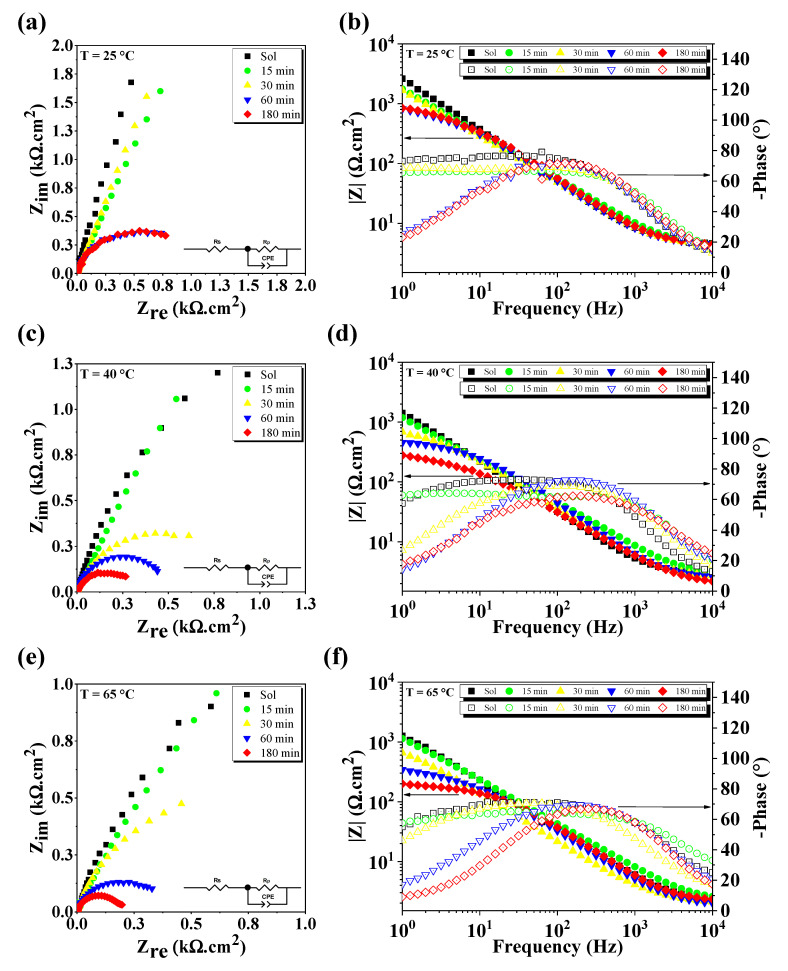
EIS results at: (**a**,**b**) 25 °C, (**c**,**d**) 40 °C, and (**e**,**f**) 65 °C.

EIS tests were used to investigate the electrode–electrolyte interface (Figure 6a–f). Electrochemical impedance spectra are shown in Figure 6a,c,e. The spectra were fitted using a Randles circuit, ($R_{S}\;\left(R_{P}CPE\right)$), as inserted in Figure 6a,c,e. Nyquist results show that there was a decrease in the diameter of the depressed impedance arc, regardless of the temperature. These changes are also clear in the Bode phase plot where by increasing the temperature and the amount of sigma phase, the impedance value (|Z|) (Figure 6b,d,f) at the frequency of 1 Hz decreased from 3.0 to 0.8 kΩ cm^2^ (25 °C), 1.5 to 0.3 kΩ cm^2^ (40 °C), and 1.25 to 0.2 kΩ cm^2^ (65 °C). This behavior can be associated with a decrease in the impedance module, indicating a decrease in corrosion resistance. In general, an increase in the impedance arc indicates a decrease in the corrosion rate of the electrolyte–electrode system [21]. The impedance arc radius decreased with increasing thermal aging time (charge transfers through the passive film with ease). In the high frequencies (close to 10 kHz), the electrolyte impedance values decreased for all specimens due to the temperature effect, characterizing a system that consists of only the resistance of electrolytes and decreased impedance of passive films [43].

Modeling the impedance of more complex real systems requires the use of equivalent circuits with constant phase elements (CPEs). The CPE has a fixed phase-shift angle and its impedance is described by Equation (1):

(1) $$Z_{CPE}=P^{-1}{\left(j\omega \right)}^{-n}$$

where $Z_{CPE}$ is the CPE’s impedance, ω is the angular frequency, j is the imaginary unit, P is the proportionality factor, n is the phase shift, $j={\left(-1\right)}^{1/2}$, and ω=2πf, where f is the frequency (Hz). The factor n depends on the surface morphology and is the deviation parameter (−1≤n≤1), where −1 is characteristic for an inductance, 1 corresponds to a capacitor, and 0 corresponds to a resistor [44].

For a circuit including a CPE, the double-layer capacitance $\left(C_{Pass}\right)$ can be calculated according to Equation (2) [45]:

(2) $$C_{Pass}=P^{1/n}R_{ct}^{\left(1-n\right)/n}$$

where $R_{ct}$ represents the charge transfer resistance. In the proposed equivalent circuit, $R_{s}$ stands for the resistance of the electrolyte between the working electrode and the reference electrode, while the element $R_{p}$ is the polarization resistance. CPEs are often used to replace the capacitance, giving a more accurate fit to the impedance data. It is related to the capacity of the material surface area of complex surface roughness, inhomogeneous reaction rates on a surface, and non-uniform current distribution [46]. All values obtained for $R_{s}$, $R_{p}$, and $C_{Pass}$ are listed in Table 3.

Table 3 shows that $R_{p}$ decreased with increasing amount of sigma phase, and its value was affected by temperature. The greater the $R_{p}$, the greater the resistance of the material to corrosion [47]. Moreover, the increase of the $C_{Pass}$ value may be related to a worse stability of the passive film on the electrode surface [43,48].

The decrease in corrosion resistance is directly attributed to the formation of the sigma phase, as reported in previous work [49,50,51]. However, the corrosion performance of microstructures containing a secondary austenite with a decrease in corrosion resistance has been reported in some studies [52]. Therefore, for a more complete study of the corrosion resistance of duplex stainless steel, the effects of each secondary phases must be taken into account [37].

This work stands out from other studies due to the possibility of studying crystallographic changes by means of EBSD and, therefore, understanding the effects on the mechanical properties and electrochemical corrosion behavior of duplex stainless steel.

## 4. Conclusions

In summary, the structural and morphological characterization proved the presence of the sigma phase with net-like shapes, and its precipitation occurred regularly and by eutectoid decomposition of ferrite, as evidenced by EBSD. Sigma phase content increased as aging treatment time increased, and it was proved that short-term aging can significantly deteriorate material ductility. EIS indicated that the corrosion resistance of DSS decreased with increases in both the sigma phase content and the measurement temperature. Therefore, EBSD analysis combined with EIS tests was found to be a successful approach to assess the decrease in corrosion resistance of DSS with secondary phases.

## Figures and Tables

**Table 1:** Normalized area fraction of phases measured by EBSD.

Phase	Solubilized	15 min	30 min	60 min	180 min
*% α*	37.5 ± 0.7	35.6 ± 2.2	31.4 ± 1.3	27.8 ± 0.6	17.2 ± 0.6
*% γ*	62.5 ± 0.7	64.0 ^(^*^)^ ± 2.2	67.7 ^(^*^)^ ± 1.1	69.3 ^(^*^)^ ± 0.9	73.7 ^(^*^)^ ± 0.8
*% σ*	n/a	0.4 ± 0.1	0.9 ± 0.2	1.7 ± 0.1	6.8 ± 0.3
*% χ*	n/a	n/a	n/a	1.2 ± 0.1	2.3 ± 0.3

n/a: not available; (*): Secondary austenite included.

**Table 2:** Summary of mechanical properties.

Specimen	(HV_0.2_)	*σ_u_* (MPa)	*σ_y_* (MPa)	*ε* (%)	(%RA)
Solubilized	257 ± 0.5	993 ± 2.8	507 ± 1.5	34 ± 1.2	57 ± 1.3
15 min	266 ± 3.1	981 ± 3.1	507 ± 2.9	34 ± 1.7	49 ± 0.7
30 min	271 ± 2.6	1001 ± 2.5	558 ± 1.3	32 ± 0.8	37 ± 1.2
60 min	287 ± 0.7	977 ± 2.7	561 ± 2.2	29 ± 1.5	25 ± 0.6
180 min	305 ± 2.7	946 ± 3.3	561 ± 1.6	22 ± 1.4	15 ± 0.7

**Table 3:** EIS Fitting parameters.

**Test Results at 25 °C**				
**Specimen**		CPE **Parameters**		
	$R_{s}$ **(Ω cm^2^)**	$C_{Pass}$ **(µF cm^−2^)**	n	$R_{p}$ **(kΩ cm^2^)**
Solubilized	3.14	4.31	0.85	21.11
15 min	2.35	6.84	0.75	12.88
30 min	1.92	7.91	0.77	9.74
60 min	1.06	8.77	0.78	0.84
180 min	0.98	10.84	0.77	0.84
**Test Results at 40 °C**				
**Specimen**		CPE **Parameters**		
	$R_{s}$ **(Ω cm^2^)**	$C_{Pass}$ **(µF cm^−2^)**	n	$R_{p}$ **(kΩ cm^2^)**
Solubilized	4.36	8.24	0.86	3.50
15 min	2.86	12.84	0.73	3.49
30 min	0.68	11.21	0.78	0.73
60 min	0.54	12.23	0.82	0.40
180 min	0.48	20.75	0.72	0.26
**Test Results at 65 °C**				
**Specimen**		CPE **Parameters**		
	$R_{s}$ **(Ω cm^2^)**	$C_{Pass}$ **(µF cm^−2^)**	n	$R_{p}$ **(kΩ cm^2^)**
Solubilized	2.37	10.97	0.81	3.08
15 min	1.84	12.71	0.71	2.27
30 min	0.53	16.75	0.79	1.07
60 min	0.41	17.45	0.77	0.32
180 min	0.31	41.78	0.78	0.16

## Supplementary Materials

The following are available online at https://www.mdpi.com/1996-1944/13/23/5511/s1, Figure S1: 2D and 3D surface profiles of the specimens (0–180 min, respectively).
