# Supplementary material for: The Effect of Microstructural Changes on Mechanical and Electrochemical Corrosion Properties of Duplex Stainless Steel Aged for Short Periods
Source: Materials (Basel). 2020 Dec 3;13(23):5511. doi: 10.3390/ma13235511 (PMC7730082; doi:10.3390/ma13235511)
Supplement: Supplementary file 1 [file materials-13-05511-s001.pdf]

materials

Supplementary Material

The Effect of Crystallographic Changes on Mechanical and Electrochemical Corrosion Properties of Duplex Stainless Steel Aged for Short Periods

David D. S. Silva *, Lindolpho S. D. C. Lima, Allan J. M. Araújo, Vinícius D. Silva, Rafael A. Raimundo, Igor Z. Damasceno, Thiago A. Simões and Rodinei M. Gomes

2D and 3D Surface Roughness Profiles

Figure S1a–e depicts the 2D and 3D surface roughness profiles of the specimens before EIS tests. The surface roughness is described by the arithmetic mean value and is based on the average length between the peaks and valleys. The quantitative parameters based on 2D line roughness, such as arithmetic mean deviation of the roughness profile (Ra), as well as the 3D surface profile parameters, such as arithmetic mean height (Sa) of the specimens were calculated according to ISO 4287 [1] and ISO 25178 [2]. The specimens (independent of the thermal aging time) showed Ra values ranging from 0.135 μm to 0.156 μm and Sa values ranging from 0.156 μm to 0.164 μm. Thus, the results show that the specimens have a similar pattern in terms of roughness.

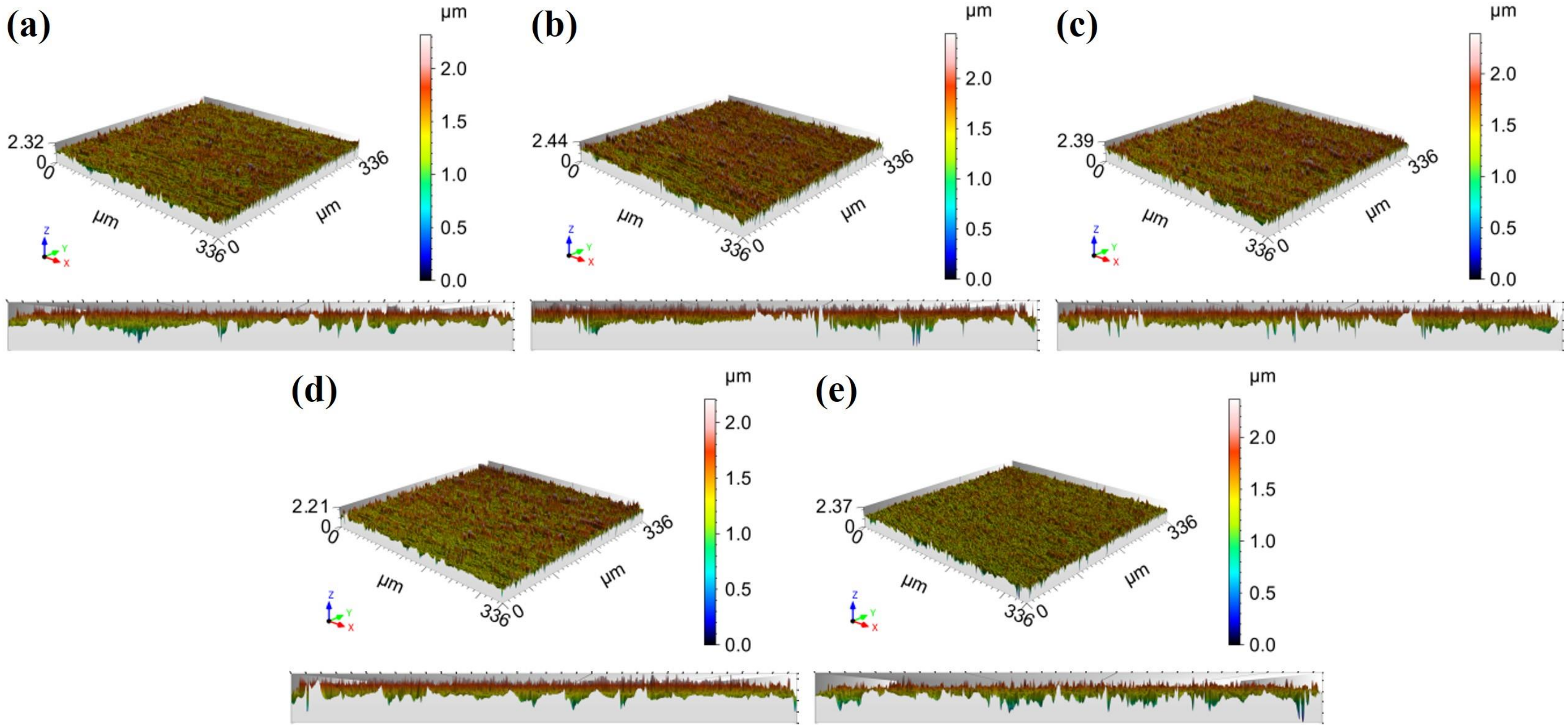

Figure S1. 2D and 3D surface roughness profiles of the specimens before the electrochemical tests in the following conditions: (**a**) solubilized and aged for: (**b**) 15 min, (**c**) 30 min, (**d**) 60 min and (**e**) 180 min.

References

1. *ISO 4287-02: Geometrical Product Specifications (GPS)—Surface texture: profile method—Terms, definitions and surface texture parameters*; International Organization for Standardization: Geneva, Switzerland, 2002.
2. *ISO 25178-2: Geometrical product specifications (GPS)—Surface texture: Areal—Part 2: Terms, definitions and surface texture parameters*; International Organization for Standardization: Geneva, Switzerland, 2012.
